# Hepatic Transcriptome Analysis Reveals Genes, Polymorphisms, and Molecules Related to Lamb Tenderness

**DOI:** 10.3390/ani13040674

**Published:** 2023-02-15

**Authors:** Kasita Listyarini, Cece Sumantri, Sri Rahayu, Md. Aminul Islam, Syeda Hasina Akter, Muhammad Jasim Uddin, Asep Gunawan

**Affiliations:** 1 Graduate School of Animal Production and Technology, Faculty of Animal Science, IPB University, Bogor 16680, Indonesia; 2 Department of Animal Production and Technology, Faculty of Animal Science, IPB University, Bogor 16680, Indonesia; 3 Immunogenomics and Alternative Medicine (IAM) Laboratory, Department of Medicine, Bangladesh Agricultural University, Mymensingh 2202, Bangladesh; 4 Faculty of Veterinary Science, Bangladesh Agricultural University, Mymensingh 2202, Bangladesh; 5 School of Veterinary Medicine, Murdoch University, Murdoch, WA 6150, Australia; 6 Center for Biosecurity and One Health, Harry Butler Institute, Murdoch University, Murdoch, WA 6150, Australia

**Keywords:** sheep, meat quality, next generation genome sequencing, single nucleotide polymorphism, genetic marker

## Abstract

**Simple Summary:**

Tenderness influences repurchase decisions of sheep meat because it is a significant factor contributing to eating satisfaction and consumer acceptance. This study analyzed the transcriptome of five high- and five low-lamb tenderness samples. The result showed potential candidate hepatic genes and polymorphisms affecting lamb tenderness. These potential candidate genes and genetic markers could be used in lamb tenderness selection programs.

**Abstract:**

Tenderness is a key meat quality trait that determines the public acceptance of lamb consumption, so genetic improvement toward lamb with higher tenderness is pivotal for a sustainable sheep industry. However, unravelling the genomics controlling the tenderness is the first step. Therefore, this study aimed to identify the transcriptome signatures and polymorphisms related to divergent lamb tenderness using RNA deep sequencing. Since the molecules and enzymes that control muscle growth and tenderness are metabolized and synthesized in the liver, hepatic tissues of ten sheep with divergent phenotypes: five high- and five low-lamb tenderness samples were applied for deep sequencing. Sequence analysis identified the number of reads ranged from 21.37 to 25.37 million bases with a mean value of 22.90 million bases. In total, 328 genes are detected as differentially expressed (DEGs) including 110 and 218 genes that were up- and down-regulated, respectively. Pathway analysis showed steroid hormone biosynthesis as the dominant pathway behind the lamb tenderness. Gene expression analysis identified the top high (such as *TP53INP1*, *CYP2E1*, *HSD17B13*, *ADH1C*, and *LPIN1*) and low (such as *ANGPTL2*, *IGFBP7*, *FABP5*, *OLFML3*, and *THOC5*) expressed candidate genes. Polymorphism and association analysis revealed that mutation in *OLFML3*, *ANGPTL2*, and *THOC5* genes could be potential candidate markers for tenderness in sheep. The genes and pathways identified in this study cause variation in tenderness, thus could be potential genetic markers to improve meat quality in sheep. However, further validation is needed to confirm the effect of these markers in different sheep populations so that these could be used in a selection program for lamb with high tenderness.

## 1. Introduction

Meat quality refers to a variety of meat characteristics such as compositional quality (lean to fat ratio, drip loss, pH) and palatability factors such as visual appearance, smell, firmness, juiciness, tenderness, and flavor [1,2]. Notably, meat tenderness is a critical characteristic since tenderness influence consumer purchase decisions [1]. Thus, improvement of sheep meat tenderness is critical for the market development and sustainability of the sheep industry. Tenderness is based on the ease of chewing that is contributed to by many factors, including muscle factors, growth performance, dietary supplementation and genetic makeup of animals [3]. Tenderness is measured mechanically by ‘shear force’ using a Warner–Bratzler shear device (WBS), where higher shear force value indicates low tenderness of meat. Meat having a ‘shear force value’ greater- and less-than 4.6 kg/cm^2^ are defined as low- and high-tenderness, respectively [4]. Though tenderness is crucial for consumer acceptance to lamb, it is worth mentioning that the trait is very difficult to predict. Tenderness is a complex trait and mainly affected by the muscle texture, amount, and solubility of connective tissues like fibrillar collagen, elastin, perimysium, and hydroxyproline, amount and composition of intramuscular fat (IMF) or marbling, contractile state of muscle fibers, and proteolysis extent of rigor muscle [3,5,6]. Furthermore, tenderness is essentially affected by some biochemical processes that occur in slaughtering, fabricating, and storing of carcasses, as well as by genomic architecture of animals [3,6]. Meat quality traits including meat tenderness, color, mineral content, and muscle oxidative capacity are generally found to be moderately heritable. The heritability varies from 0.15 to 0.30 [7], indicating that selection will be efficient in enhancing the genetic merit of meat quality traits. It is widely accepted that genetic improvement through breeding is a feasible approach for improving meat quality traits. Likewise, tenderness is moderately heritable (*h*^2^ = 0.25) and could be improved through selection [8]. Identification of genetic factors controlling meat quality traits including tenderness is the foremost step in order to implement a breeding program. Several potential genes for meat quality attributes have been found, including the *CYP2A6* and *KIF12* genes for flavor and odor [9], *APOA5* for fatty acids (FA) composition [10], *CAST* and *GDF-8* genes for tenderness [11].

Quantitative trait loci (QTL) for meat quality traits, including tenderness have been identified on chromosome OAR 18 in crossbred sheep [12]. QTL on OAR 1, 3, 6, and 24 are reported to be associated with muscle, fat, and bone traits [13]. A genome wide association study (GWAS) using the Illumina 600k (HD) and 50k Ovine SNP chips were applied to identify markers including those associated with sheep tenderness [14]. Bolormaa et al. [14] reported QTL that influence meat tenderness, color, myoglobin, glycogen, and omega-FA, and decrease long chain saturated FA, which are highly valuable in a selection program. However, more study, especially high throughput sequencing (HTS) or deep sequencing transcriptome (RNA-Seq) analysis should be implemented to unravel the genetic architecture of these traits [15]. RNA-Seq is an advanced method of transcriptome profiling that uses deep-sequencing technologies to provide insight into the transcriptome of the host [16]. RNA-Seq is successfully facilitating the discovery of novel transcripts, identification of gene polymorphisms (single nucleotide polymorphisms, SNPs), alternatively spliced genes, and detection of allele-specific expression [16]. Recently several studies have effectively employed the RNA-Seq technology to explore DEGs controlling meat quality traits in sheep including flavor/odor [17], FA composition [18], and meat quality traits in different muscles [19].

Meat tenderness is determined by the intrinsic characteristics of meat, specifically the amount of glycogen [20]. In sheep, the liver is the most significant glucose-producing organ, producing 85–90% of the body’s total glucose production [21]. Therefore, muscle glycogen in lambs fed a diet high in roughage will have come from liver-produced glucose. Glycogen is stored in the liver to maintain blood glucose concentration and homeostasis [22]. Furthermore, liver glycogen concentration is reported to affect muscle characteristics and is positively correlated with muscle glycogen [23,24]. However, post-mortem glycolic changes affect the physical and sensory features of meat quality traits such as pH, color, tenderness, and cooking loss [25]. The post-mortem breakdown of muscle glycogen yields lactic acid, the accumulation of which contributes to the change in meat pH [26]. Note, the antemortem glycogen level in muscle is positively correlated to the glycogenesis in the liver [21,22]. A relationship between glycogen depots in liver and muscle and ultimate muscle pH values has been described postulating that higher glycogen storages contribute to the lower ultimate pH in the muscle [27,28]. Furthermore, all dietary supplements and muscle component-associated molecules are being metabolized in the liver; thus it is pivotal to unravel the hepatic transcriptome affecting meat quality traits including meat tenderness. However, application of RNA-Seq to identify DEGs and polymorphisms affecting lamb tenderness is limited. Hence, the objective of this study is to decipher the transcriptome and polymorphisms within the liver with divergent tenderness in Indonesian sheep using Illumina Hiseq 2500. Several candidate genes and genetic markers related to the tenderness of sheep meat were identified which could contribute to a sustainable sheep industry by improving meat quality.

## 2. Materials and Methods

### 2.1. Animals and Phenotype

Tissue samples (*longissimus dorsi*, *semitendinosus*, liver tissues) and phenotypes were collected from the male Garut composite sheep (GCS) with an average liveweight of 30 kg and age of 12 months. GCS sheep are 50% indigenous Garut sheep, 25% St. Croix sheep from the Virgin Islands, and 25% Moulton Charolais sheep from France [29] (Supplementary Figure S1). All the sheep (*n* = 140) were slaughtered at PT Pramana Pangan Utama, IPB University. The Animal Ethics Commission of the IPB University approved all procedures involving animals (approval no. 117-2018 IPB). Phenotypes were measured for meat quality traits including tenderness (shear force), water holding capacity (WHC), pH, and cooking loss. Meat tenderness was measured using Warner–Bratzler shear force (WBSF) [30]. The WHC was measured by measuring the amount of water lost (mgH_2_O). WHC is the percentage of weight lost from 5 g meat samples after being pressurized at 2250 g for 5 min [31]. The pH value was measured with a pH meter after carcass being stored for 24 h postmortem (final pH). Cooking loss was measured by deducting the initial weight of the sample meat after being cooked in a water bath at a temperature of 80 °C for 1 h [30].

For the RNA sequencing analysis, ten GCS were selected from the pool of 140 sheep with extreme divergent tenderness phenotypes. The average shear force value for high (HT) and low (LT) tenderness groups were 3.14 ± 0.09 and 4.69 ± 0.67, respectively (Table 1). RNA was extracted from the livers of 5 sheep with extremely high (HT) and 5 sheep with extremely low (LT) tenderness levels using the RNeasy Mini Kit (Qiagen, Hilden, Germany).

### 2.2. Library Construction and Sequencing

A Nanodrop 2000 Spectrophotometer was used to measure the quantity of RNA and an Agilent 2100 Bioanalyzer was used to measure the quality of RNA (Agilent Technologies, Sangta Clara, CA, USA). TruSeq RNA Library Prep Kit v2 was used to prepare libraries from RNA samples of acceptable quality and quantity with a minimum RNA Integrity Number (RIN) > 7.0 (Illumina, San Diego, CA, USA). A total of 101 base paired-end sequencing of the 10 samples were performed using Illumina HiSeq-2500 Platform (Illumina) and sequencing reads were mapped to the sheep (*Ovis aries*) reference genome Oar_v4.0. The sequencing information was delivered to NCBI (Accession: PRJNA847713, ID: 847713).

### 2.3. Differential Gene Expression and Pathway Analysis

We performed differential expression analysis to assess the gene expression differences between two distinct sample conditions. To discover significant DEGs, the absolute value of the log2 (fold change) > 1.5 was used as the threshold. The R package DESeq was used to identify DEGs with a false discovery rate (FDR) of 0.05 compared to the sheep group (criteria: fold change > 1.5 and *p* ≤ 0.05) [32]. DESeq then includes a Generalized Linear Model (GLM) function for calculating both within and between group deviations. DAVID [33] was used to perform functional annotation and pathway enrichment of DEGs. The DEGs were used for gene ontology (GO) enrichment [34] and pathway analyses in the Kyoto Encyclopedia of Genes and Genomes (KEGG) [35]. The GO terms and pathways with *p* ≤ 0.05 were considered significantly enriched, and only the genes significant in the tests (*p* ≤ 0.05) were chosen for further investigation.

### 2.4. Network Enrichment Analysis

The network enrichment analysis was carried out with the help of the open-source online tool NetworkAnalyst [36]. The literature-curated PPI database imported from InnateDB was used to build the PPI network with human orthologs of the differentially expressed genes [37]. The standard network algorithm generated 1 larger subnetwork called “continent” and 7 smaller subnetworks called “island”. Due to all the islands having only one seed gene with 3–9 nodes connected by 2–8 edges, they were given additional consideration. The continent was further modified for better visualization by using the tool’s ‘minimize network’ function. The PPI was represented as nodes (circles representing regulatory genes) linked by edges (lines representing the direct molecular interactions). For detecting the network’s highly interconnected genes (hub genes), two network centrality measures were used: degree (number of connections to other nodes) and betweenness (number of shortest paths passing through the node). Higher degree and betweenness nodes were regarded as potentially more important network hubs in cellular signal trafficking. Furthermore, liver-specific co-expression networks were created by incorporating the TCSBN database [38] into the NetworkAnalyst tool.

### 2.5. Analysis of Quantitative Real-Time PCR (qRT–PCR) Validation

Reverse transcriptase PCR was performed by transcribing extracted RNA into complementary DNA (cDNA) using a First Strand cDNA Trancriptor Synthesis (Thermo Scientific, Vilnius, Lithuania) kit based on the manufacturer’s protocol. Quantification of cDNA was performed by a qRT–PCR method with AG qTower 4 channel (Analytic Jena engine, Jena, Germany). The online tool Primer3 software (https://primer3.ut.ee/, accessed on 1 March 2021) [39] was used to design gene specific primers for qRT–PCR (Table 2). The 96-well microtiter plate in each run contained one cDNA sample and no template control. Each sample was examined twice (technical replication), and the geometric mean of the Ct values was applied to profile mRNA expression. For normalization of the target genes, the geometric mean of two housekeeping genes, β-Actin and GAPDH was used. Ct values were calculated by subtracting the targeted gene from the geometric mean of the reference genes: ∆Ct = Ct_target_ − Ct_housekeeping_ genes [40].

### 2.6. Analysis of Gene Variation

SNP calls were made on the mapping files generated by the TopHat algorithm for gene variation analysis using the ‘samtools mpileup’ command and associated algorithms [41]. For further analysis, we chose variants with a minimum Root Mean Square (RMS) mapping quality of 20 and a minimum read depth of 100. The selected variants were compared to the dbSNP database to identify previously studied polymorphisms. In order to identify differentially expressed genes with sequence polymorphisms, we cross-checked and filtered these variants based on their chromosomal positions against DEGs, retaining only those variants that mapped to DEG chromosomal positions. In this way, we could pick a few mutations that mapped to DEGs among thousands of identified potential sequence polymorphisms. Furthermore, we calculated the read/coverage depth of these polymorphisms in all samples to determine whether they were segregated in only one sample group (high or low tenderness) or in both groups (high and low tenderness). Using the GeneWise software (http://www.ebi.ac.uk/Tools/psa/genewise/, accessed on 20 April 2021), the identified SNPs were grouped as synonymous or non-synonymous by comparing protein sequences and nucleotide incorporated SNP position [42].

### 2.7. SNP Validation and Association Study

A SNP in each of three highly polymorphic DEGs, and genes known to play roles in tenderness (*OLFML3*, *ANGPTL2*, and *THOC5*) was chosen for validation (Table 2). The muscle (*Longissimus dorsi*) samples from 140 sheep were collected for DNA extraction until a final concentration of 50 ng/mL DNA was obtained. For genotyping, the PCR–RFLP (Polymerase Chain Reaction–Restriction Fragment Length Polymorphism) method was used. The PCR was carried out in a 15 µL volume with 1 µL of genomic DNA, 0.4 µL of primers, 6.1 µL of MyTaq HS Red Mix, and 7.5 µL of nuclease water. A 1.5% agarose gel (Fischer Scientific Ltd., Meridian, MS, USA) was used to examine the PCR product, which was then digested with the appropriate restriction enzyme. The digested PCR-RFLP products were resolved in 2% agarose gels. PROC GLM in SAS 9.2 was used to calculate the effect of genotypes on meat quality traits (SAS Institute Inc, Cary, NC, USA). One-way analysis of variance (ANOVA) followed by Duncan’s test was used to compare the loci genotypes’ least square mean values.

Y*_i_* = *μ* + genotype*_i_* + e*_i_*

where: Y*_i_* = the meat quality trait; *µ* = the population mean; genotype*_i_* = the fixed effect of *i*-th genotype; e*_i_* = the residual error.

## 3. Results

### 3.1. Phenotype of Meat Quality Traits in Sheep

The phenotypic traits of meat quality measured were: pH, shear force (for tenderness), cooking loss, and WHC. Descriptive parameters for meat quality traits’ data are given in Table 1. The average pH, shear force, cooking loss, and WHC values were 5.98, 3.66, 46.46, and 28.09, respectively. Sheep having a ‘shear force value’ greater and less than 4.6 kg/cm^2^ were defined as low- and high-meat tenderness, respectively [4].

### 3.2. Overview of the RNA Deep Sequencing Data

In this study, cDNA libraries have been sequenced from ten liver tissues collected from phenotypically divergent (five HT, and five LT) sheep using Illumina HiSeq 2500. The sequencing produced sequence read clusters with a maximum of 100 bp. The total number of reads after quality control and filtering ranged from 20.02 to 21.90 million. The total number of reads in each group, as well as the number of reads that have been mapped to reference sequences are given in Table 3. In the LT group, 87.26% to 88.78% of total reads were aligned to reference sequences, whereas 83.85% to 88.80% of total reads were aligned to reference sequences in case of HT group (Table 3).

### 3.3. Differential Gene Expression Analysis

**Figure 1:**
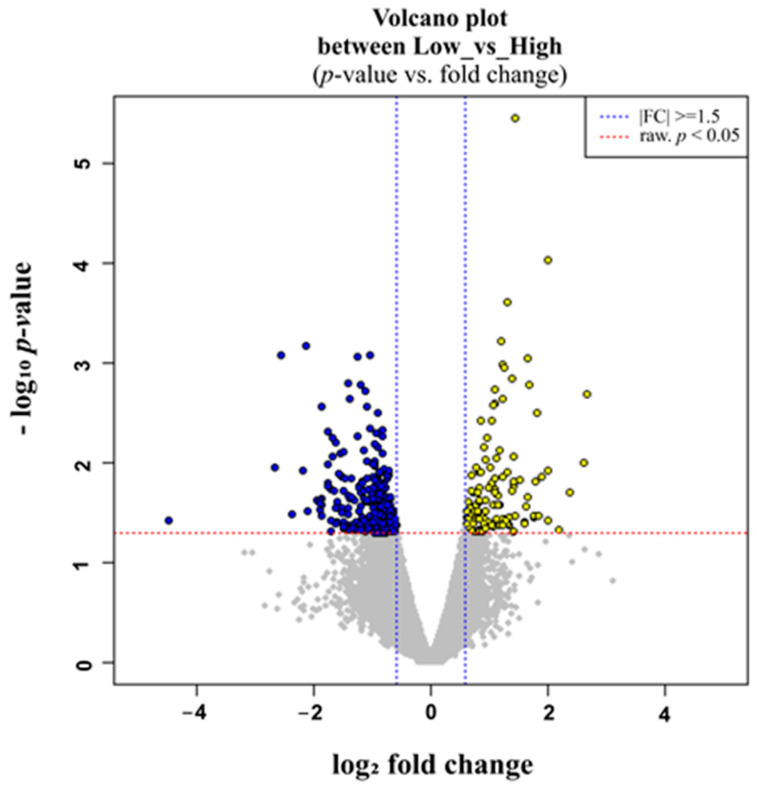
Volcano plot. The 110 differentially expressed protein coding genes (represented in blue) were plotted as a volcano with a fold change of 1.5 and a *p*-value of 0.05. The *x*-axis values are the base mean expression values, and the *y*-axis values are the log2 expression values (fold change).

**Figure 2:**
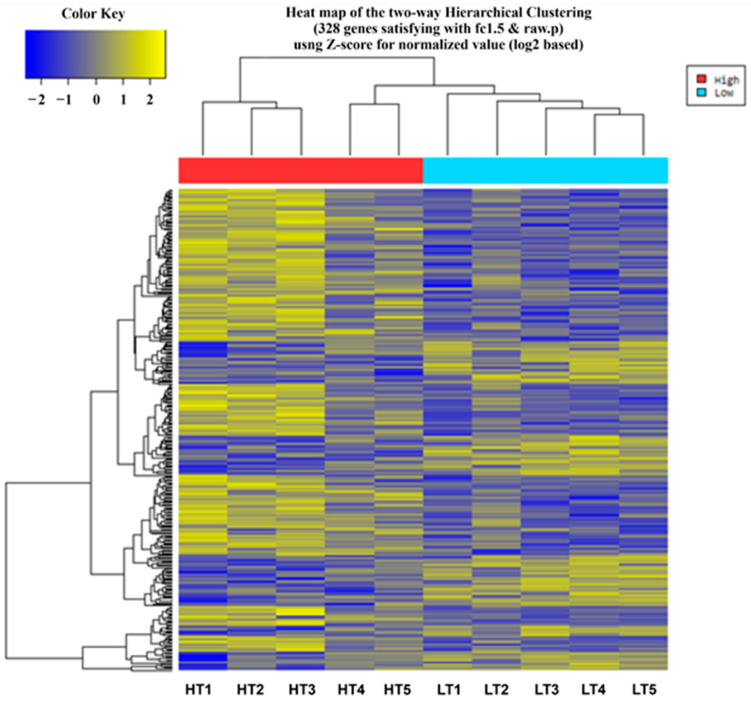
A heatmap depicting the expression of hepatic genes in sheep. The red blocks are overexpressed genes, while the green blocks are underexpressed genes. HT 1–5 sheep have high meat tenderness, and LT 1–5 sheep have low meat tenderness.

The raw reads of DEGs in the liver tissues of sheep with HT and LT levels were calculated using the R package DESeq. To identify DEGs in the liver with divergent (high and low) tenderness, a negative binomial distribution-based method implemented in DESeq was used. The differential expression analysis yielded 328 hepatic DEGs using the criteria *p* adjusted 0.05 and log2 fold change > 1.5 (Figure 1). A total of 110 and 218 genes were identified as up- and down-regulated, respectively, in the HT and LT groups (Supplementary Table S1). The log2 fold change values for DEGs ranged from 4.09 to 4.80. Heatmaps (Figure 2) depicted the top 30 up- and down-regulated hepatic genes found in sheep with high and low tenderness. The top 30 up- and down-regulated genes, along with log FC and *p*-values are presented in Supplementary Table S2. The differential expression analysis of these data indicated both novel transcripts and genes previously reported in other gene expression studies (Supplementary Table S1).

### 3.4. Functional Analysis

**Figure 3:**
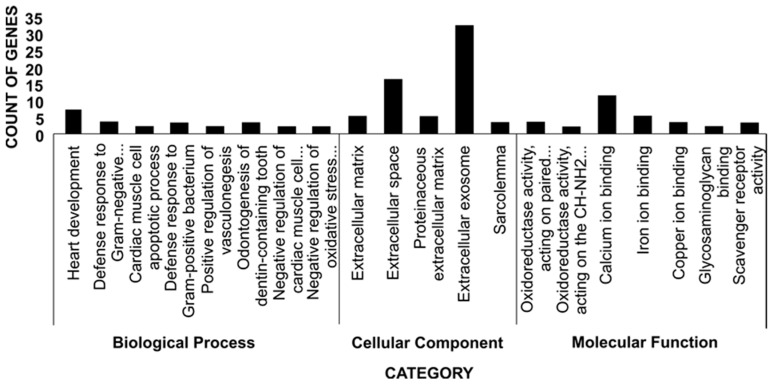
Illustration of a histogram of GO classification in Garut composite sheep. The findings were divided into three categories: biological process, cellular component, and molecular function. The number of genes involved is indicated on the *y*-axis.

**Figure 4:**
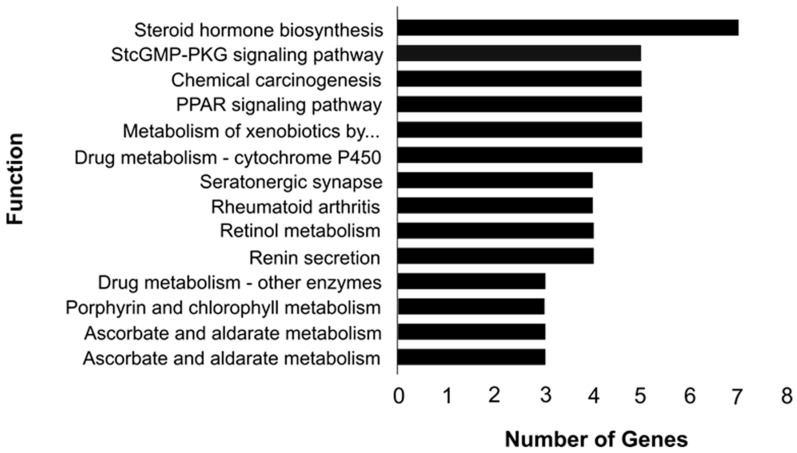
KEGG pathway histogram illustration in Garut composite sheep. The number of genes involved is indicated on the *x*-axis.

Cellular components, molecular functions, and biological processes were the most important GO terms discovered (Figure 3). Calcium ion binding and iron ion binding were the molecular functions that controlled the metabolism of tenderness-related molecules. The cellular processes identified were mainly related to extracellular exosome and extracellular space. The biological mechanisms revealed were correlated to heart development and defense response to Gram-negative bacteria (Table 4). The DAVID tool identified KEGG pathways that were overrepresented for DEG. The dominant pathway for differences in lamb tenderness level was steroid hormone biosynthesis (Figure 4). The hepatic genes defined in these pathways with high and low tenderness levels are shown in Table 5.

### 3.5. The Hepatic Transcriptome Network’s Regulatory Hub Genes

**Figure 5:**
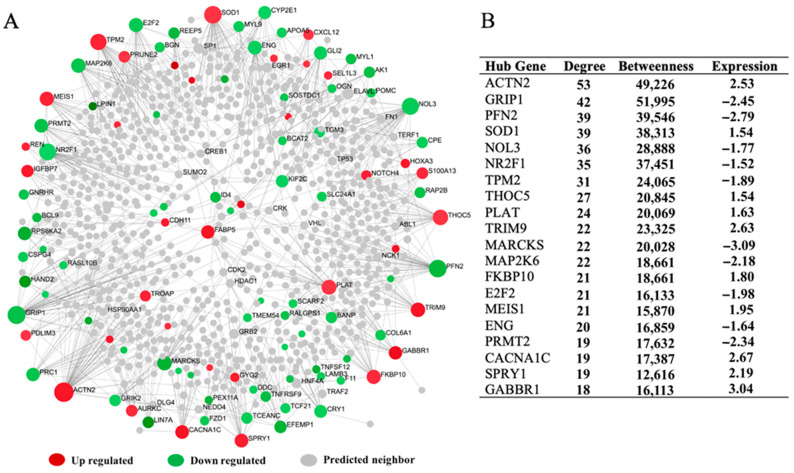
Protein–protein interaction network of hepatic transcriptomes from Garut composite sheep with divergent tenderness. (**A**) The PPI network showing the hub genes and their connections. (**B**) The table illustrates the network centrality measures (degree and betweenness) and expression values of potential hub genes.

**Figure 6:**
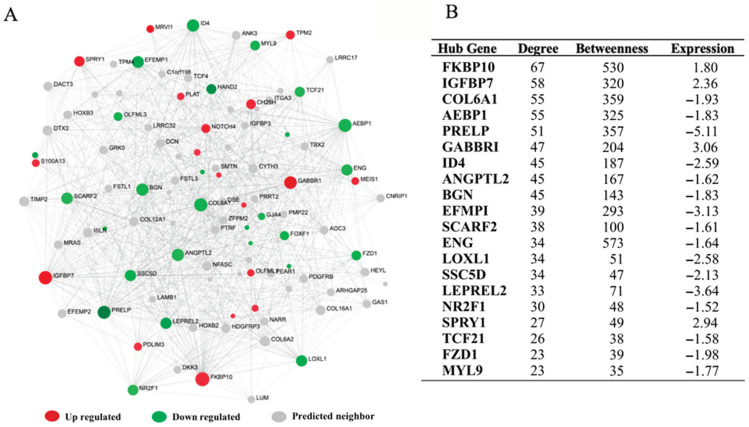
Co-expression network of hepatic transcriptomes from Garut composite sheep with divergent tenderness. (**A**) The co-expression network showing the hub genes and their connections. (**B**) The table illustrated the network centrality measures (degree and betweenness) and expression values of potential hub genes.

A PPI network with 117 seed genes and 944 nodes connected by 1138 edges was built to identify potential regulatory hub genes in the hepatic transcriptional network. The potential hub genes were identified using network centrality measures, with *ACTN2*, *SOD1*, *TPM2*, *THOC5*, *PLAT*, *TRIM9*, *FKBP10*, *MEIS1*, *CACNA1C*, *SPRY1*, and *GAAR1* up-regulated, and *GRIP1*, *PFN2*, *NOL3*, *NR2F1*, *MARCKS*, *MAP2K6*, *E2F2*, *ENG*, and *PRMT2* down-regulated (Figure 5A,B). In addition, a liver-specific gene co-expression network was developed to identify additional hub genes that may have been missing in the PPI network. The co-expression network revealed that the majority of the potential hub genes, including *COL6A1*, *AEBP1*, *PRELP*, *ANGPTL2*, *EEEMP1*, *SCARF2*, *ENG*, *LOX1*, and *SSC5D*, were downregulated, while only four hub genes (*FKBP10*, *IGFBP7*, *GABBR1*, and *SPRY1*) among the top twenty were upregulated in the liver tissue obtained from GCS (Figure 6A,B). Surprisingly, the common hub genes in both the PPI and the co-expression network were *FKBP10*, *GABBR1*, *ENG*, *NR2F1*, and *SPRY1* (Supplementary Tables S3 and S4).

### 3.6. Quantitative Real-Time PCR Validation of Selected DEGs (qRT–PCR)

**Figure 7:**
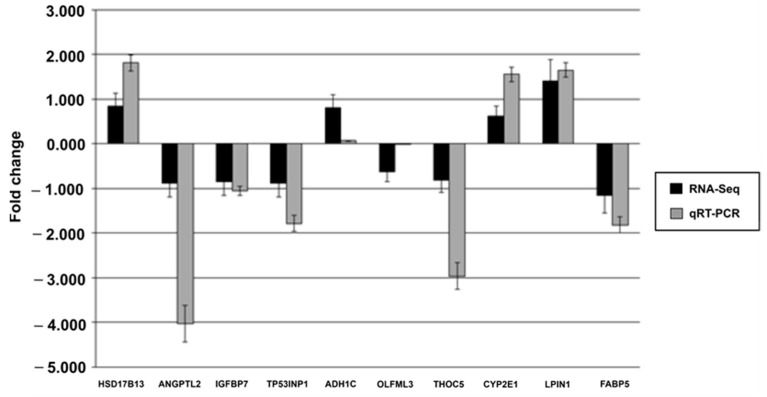
Validation of ten hepatic DEGs in sheep with varying meat tenderness using qRT–PCR. The validation was carried out using the same RNA samples as the RNA deep sequencing.

To validate the RNA-Seq results, a total of ten genes (*HSD17B13*, *ANGPLT2*, *IGFBP7*, *TP53INP1*, *ADH1C*, *OLFML3*, *THOC5*, *CYP2E1*, *LPIN1*, and *FABP5*) were chosen and quantified using qRT–PCR. The same samples that were used for deep sequencing were used for this purpose. A comparison of qRT–PCR data for ten selected genes revealed quantitative expression concordance with RNA-Seq results (Figure 7). The qRT–PCR gene expression values were normalized using two housekeeping genes (*GAPDH* and *β-Actin*). Detailed GenBank accession numbers, primer sequences, and annealing temperatures for qRT–PCR used in this study are provided in Table 2.

### 3.7. Analysis of Gene Variation and an Association Study

**Figure 8:**
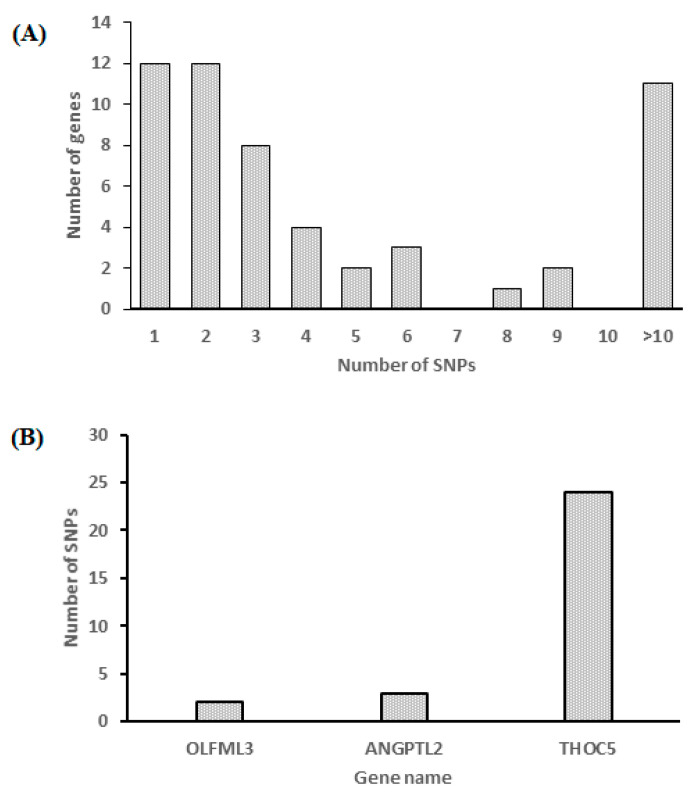
The distribution of the number of SNPs found in DEGs. (**A**) Number of SNPs; (**B**) Gene Name.

In 54 DEGs with high and low tenderness, 334 single nucleotide polymorphisms (SNPs) were found (Supplementary Table S5). The selected polymorphisms identified in hepatic DEGs are listed in Supplementary Table S6. Figure 8A,B show the distribution of the number of genes with SNPs and the selected SNPs used for validation. Furthermore, three SNPs were chosen for association analysis based on gene functions related to tenderness (Figure 8B and Supplementary Table S6). The selected SNPs were found in the genes *OLFML3*, *ANGPTL2*, and *THOC5*. Note, the SNP in *OLFML3* and *ANGPTL2* genes are located in intron 4 and 5, respectively, whereas the SNP in *THOC5* gene is located in exon 17. The segregation and association of these SNPs in the sheep population (*n* = 140) used in this study were validated. The association analysis suggested that the polymorphisms in *OLFML3*, *ANGPTL2*, and *THOC5* were associated (*p* < 0.05) with tenderness (Table 6) in the sheep population.

## 4. Discussion

### 4.1. Analysis of RNA Seq Data

Transcriptome profiling sheds light on the genetics underlying tenderness in sheep. Hence, this comparative RNA-Seq study involving divergent (high vs. low) tenderness in GCS was performed. The identified DEGs determine the functional complexity of tenderness and provide important information on phenotypic and functional differences in tenderness in lamb. The mapping results showed that the average number of reads was 20.74 million, with 87.27% of the reads classified as mapped reads corresponding to exon reads (Table 3). The percentage of mapped reads was higher than the previous study by Gunawan et al. [17] (85.73%) and Gunawan et al. [18] (85.89%) in Indonesian Javanese fat-tailed sheep. The percentage of mapped reads is an indicator of the overall sequencing accuracy and absence of contaminating DNA [43].

### 4.2. Differentially Expressed Gene Analysis

Among 328 DEGs, the differences in gene expression were more clearly shown using the top 30 genes that were highly expressed and the top 30 genes that were expressed the lowest in liver tissue with different levels of tenderness, along with FC log values and *p*-values (Supplementary Table S2). Potential candidate genes that were upregulated include *TP53INP1*, *APOA5*, *CYP2E1*, *HSD17B13*, *ADH1C*, and *LPIN1*. The *TP53INP1* gene belongs to the p53 tumor protein family which has been shown to be associated with skeletal muscle growth, myocytes’ division and maturation in pigs [44]. The *APOA5* (*Apolipoprotein A5*) gene is reported to have an influence on FA metabolism in Indonesian sheep [10]. The *CYP2E1* gene (*Cytochrome P450 2E1*) plays a key role in the enzymes’ metabolism in the liver that affects the meat flavor in pig [45]. Note, the *CYP2A6* gene was previously found to be associated with lamb flavor and odor in sheep [9]. The *HSD17β13* is a *17β-HSD* family gene that mediates the physiological functions of reproductive hormones, and the *HSD17β* gene family was reported to be associated with meat quality traits in pigs [46]. It has been reported that the *ADH1C* (*Alcohol Dehydrogenase 1C*) gene is associated with vitamin A content and muscle tenderness in Korean cattle [47]. The *Lipin 1 (LPIN-1)* gene is a key factor regulating lipid, dietary glucose, and polyunsaturated FA metabolism [48], and thus may regulate muscle tenderness.

The top down-regulated candidate genes found to influence the metabolism of muscle tenderness-related molecules were *ANGPTL2*, *IGFBP7*, *FABP5*, *CH25H*, *LOXL3*, *OLFML3*, *THOC5*, and *AEBP1*. The *ANGPTL2* gene is a member of angiopoietin-like proteins family that was reported to be associated with fat deposition in cattle [49]. Both the *AEBP1* and *IGFBP7* genes were reported to influence muscle development in pigs [50]. The *IGFBP7* (*Insulin-like growth factor binding-protein 7*) binds to *IGF* and regulates *IGF*-signaling pathways. Overexpression of the *IGFBP7* gene is reported to inhibit lipid accumulation in tissues [51], thus downregulation may positively affect the lipid accumulation and muscle tenderness because the IMF content or marbling is positively correlated with tenderness. The *FABP5* gene is a member of *FABPs* (*fatty acid-binding proteins*) family that controls lipid metabolism [52] and thus may affect muscle tenderness. Furthermore, *CH25H* (*cholesterol 25-monooxygenase*) inhibits the cholesterol biosynthetic enzymes and has a defense function [53], but its association with muscle tenderness is yet to be deciphered. *LOXL3* (*Lysyl oxidase-like 3*) is a member of the lysyl oxidase family that play roles in extracellular matrix maturation and are involved in bone development [54]. The *OLFML3* (*Olfactomedin-like 3*) gene has been reported to be differentially expressed during muscle development in pigs [55]. The *THOC5* gene is well known for playing a key role in lipid and FA metabolism in cattle [56]. The positive effect of lipids on meat tenderness might be due to the presence of lipids in the perimysium, that separates muscle fiber bundles [56]. Note, some of the important DEGs have been studied in cattle and pigs [49,50,55,56], but very little or no study has been performed in sheep with regards to meat quality traits, including tenderness.

### 4.3. Biological Function Analysis for DEGs

This study enriched the GO categories of biological processes, cellular components, and molecular functions (Figure 3 and Table 4). The enriched biological processes identified were mostly related to heart development, defense response to bacteria, positive regulation of vasculogenesis, negative regulation of muscle cell apoptotic processes, and negative regulation of the oxidative stress-induced intrinsic apoptotic signaling pathway. Oxidative and apoptotic processes are involved in metabolism of molecules that affect meat tenderness. The majority of the oxidative metabolism-related proteins are found to play a role in stress regulation too [57]. Muscles are usually exposed to a variety of reactive oxygen species resulting from oxidative stress, thus, increased antioxidant activities may regulate apoptosis and influence meat tenderness [58]. Several studies reported that biological pathways related to meat tenderness usually include proteolysis, muscular structure and contraction, oxidative stress, heat shock proteins, and apoptosis [59,60].

Cellular components identified consist of extracellular matrix, extracellular space, proteinaceous extracellular matrix, extracellular exosomes, and sarcolemma (Figure 3). The extracellular matrix provides biomechanical strength to the intramuscular connective tissues and regulates the structural properties of myocytes. Decorin and laminin are two extracellular matrix molecules that modulate the activity of myostatin, which regulates skeletal muscle mass. Furthermore, decorin has been shown to activate the insulin-like growth factor-I receptor (*IGF-IR*) and myogenic cell differentiation, and thus functions as a signaling molecule for myogenic cells. The structural integrity of the intramuscular connective tissues increases with animal growth. The collagen fibrils within the endomysium get connected, and the collagen fibers in the perimysium become increasingly thick and their wavy pattern becomes more regular during muscle development. These modifications increase the mechanical strength of the intramuscular connective tissues which contributes to meat toughening [61]. The molecular functions controlling the tenderness-related molecules’ metabolism were related to oxidoreductase activity, acting on paired donors with incorporation or reduction of molecular oxygen, reduced flavin or flavoprotein, oxidoreductase activity acting on the CH-NH2 group of oxygen, calcium ion binding, iron ion binding, copper ion binding, glycosaminoglycan binding, and scavenger receptor activity (Figure 3). Oxidoreductase activity that controls the muscle mass and strength, and calcium ion binding that regulates the muscle contractile properties along with hormones were previously identified in meat quality traits analysis in Duroc pigs [62]. Pathway analysis showed that steroid hormone biosynthesis, the PPAR signaling pathway, metabolism of xenobiotics by cytochrome P450, chemical carcinogenesis, cGMP-PKG signaling pathway, and drug metabolism—cytochrome P450 were the dominant pathways for differences in tenderness in lamb (Figure 4). Steroid hormones play critical roles during myogenesis by influencing cell differentiation [63]. The PPAR signaling pathway, which is involved in lipid metabolism, has long been recognized as an important biological pathway controlling meat quality in animals. The primary transcription regulator in PPAR signaling, peroxisome proliferator-activated receptor gamma has been reported to be a key factor in controlling the transcription of many genes involved in adipogenesis pathways [64]. PPAR signaling pathway genes influence muscle tenderness by causing phenotypic differences in marbling in livestock [65].

### 4.4. The Hepatic Transcriptome Network’s Regulatory Hub Genes

Muscle tenderness traits, like many other quantitative traits, are most likely regulated by multiple genes that interact with one another via an interconnected network. As a result, network-based approaches are thought to be more sensitive in identifying regulatory gene molecules for global transcriptome alterations [66]. Herein, PPI network and co-expression analysis was performed to scrutinize the regulatory hepatic genes in GCS with divergent tenderness. The hepatic transcriptome network’s regulatory hub genes identified several key genes, including *ACTN2*, *SOD1*, *TPM2*, *THOC5*, *PLAT*, *TRIM9*, *FKBP10*, *MEIS1*, *CACNA1C*, *SPRY1*, and *GAAR1*, which were upregulated in the liver tissue (Figure 5A,B). The *ACTN2* gene is involved in muscle fiber composition and muscle contraction [67]. *TPM2* is involved in muscle contraction, muscle development, and lipid accumulation [68]. *THOC5* gene is reported to influence lipid and FA metabolism, as well as affecting meat tenderness [56]. The potential down-regulated hub genes were identified including *GRIP1*, *PFN2*, *NOL3*, *NR2F1*, *MARCKS*, *MAP2K6*, *E2F2*, *ENG*, and *PRMT2* (Figure 5A,B). The *GRIP1* gene was previously reported to be associated with marbling [69]. SNPs in the *MAP2K6* gene are associated with marbling score, back fat thickness, and carcass weight in Hanwoo cattle [70]. *MAP2K6* belongs to the protein kinase family and regulates the mitogen-activated protein kinase pathway that controls muscle growth [71]. The *E2F2* gene plays an important role in skeletal muscle development by activating transcription factor-2 [72]. The advent of transcriptional network analyses has proved that functionally related genes are usually co-expressed in various tissue and organism. Constructing a co-expression network from transcriptome datasets has become a widely used alternative to conventional analysis method for searching highly relevant genes of complex biological function. The co-expression network identified several downregulated hub genes including *COL6A1*, *AEBP1*, *PRELP*, *ANGPTL2*, *EEEMP1*, *SCARF2*, *ENG*, *LOX1*, and *SSC5D*, whereas only four upregulated hub genes, namely *FKBP10*, *IGFBP7*, *GABBR1*, and *SPRY1* were identified in the liver tissue obtained from GCS with the divergent meat tenderness trait (Figure 6A,6B). The *AEBP1* and *IGFBP7* genes influence muscle development in pigs [50]. The *ANGPTL2* gene is reported to be associated with the fat deposition process in cattle [49]. The *IGFBP7* gene is considered a candidate gene associated with meat quality traits according to results of function and pathway analysis in crossbred sheep [73]. The *GABBR1* gene is also reported to be a candidate gene for fat deposition in the sheep tail [74].

### 4.5. Association between Candidate Markers and Phenotypes

In this study, selected polymorphisms in the *OLFML3*, *ANGPTL2*, and *THOC5* genes were revealed to be associated with meat quality traits (Table 6). The polymorphism in the *OLFML3* (C > T, g.90317673) gene was significantly (*p* < 0.05) associated with tenderness and cooking loss. The percentage of cooking loss is proportional to the shear force value. The higher the percentage of cooking loss, the higher the shear force value [75]. The polymorphism in the *ANGPTL2* (G > A, g.8930776) and *THOC5* (C > T, g.68234589) genes were significantly (*p* < 0.05) associated with tenderness. Meat tenderness is affected by the biochemical properties of muscle fibers and the connective tissue matrix, as well as by age, primarily due to cytoskeletal protein degradation. Tenderness is a key trait influencing repurchase decisions because it is a major factor that contributes to eating satisfaction and consumer acceptance [76]. The *OLFML3* gene was previously reported to be influencing meat tenderness in cattle [56]. The *THOC5* gene was also reported to affect meat tenderness [56]. A recent study has identified that the *OLFML3* gene is associated with meat quality traits including tenderness [77], however, due to fewer association studies in sheep, the scope of comparing the results is limited.

## 5. Conclusions

This transcriptome analysis using RNA deep sequencing revealed potential candidate hepatic molecules, genes, and polymorphisms affecting lamb tenderness. This study suggests several candidate genes such as *TP53INP1*, *CYP2E1*, *HSD17B13*, *ADH1C*, *LPIN1*, *ANGPTL2*, *IGFBP7*, *FABP5*, *OLFML3*, and *THOC5* that might control the metabolism of molecules involved in lamb tenderness. Furthermore, several SNPs were detected in the hepatic DEGs and associations of selected markers with tenderness were validated, such as polymorphisms in the *OLFML3*, *ANGPTL2*, and *THOC5* genes that could be potential markers for meat tenderness in sheep. However, further validation is needed to confirm the effect of these genetic markers in other sheep populations, so that they can be considered in selection for sheep with higher meat tenderness.

## Figures and Tables

**Table 1:** Descriptive statistics of meat quality composition in sheep.

Meat Quality Composition	Mean	SD	Low (*n* = 5)		High (*n* = 5)	
	*n* = 140	*n* = 140	Mean	SD	Mean	SD
pH	5.98	0.57	6.11	0.11	5.95	0.22
Tenderness *	3.66	0.76	4.69	0.67	3.14	0.09
Cooking loss (%)	46.46	8.09	47.91	6.30	49.40	2.90
Water holding capacity (%)	28.09	3.22	26.22	2.00	26.68	3.17

* The meat quality trait ‘tenderness’ is measured as ‘shear force’ with unit kg/cm^2^.

**Table 2:** GenBank Accession numbers and primer sequences for qRT–PCR and genotyping.

Gene Name	Accession Number	Primer sequence	Tm (°C)	Application	Enzymes	Size (bp)	Cutting Size (bp)
*HSD17B13*	XM_004009979.5	F: 5′-CCC ATC AAC ACC TAG AAT GC-3′ R: 5′-CAG CAG TGA TTC CAA GTA GG-3′	61	qRT–PCR	-	178	-
*ANGPTL2*	XM_027966435.2	F: 5′-TTA ATG AAT AAC CAG GGG CC-3′ R: 5′-CTG CTG AGG TAA TAG GCA CA-3′	53	qRT–PCR	-	215	-
*IGFBP7*	NM_001145181.1	F: 5′-CTG TCC TCA TCT GGA ACA AG-3′ R: 5′-TCT CCA GCA TCT TCC TTA CT-3′	56	qRT–PCR	-	169	-
*TP53INP1*	XM_042254467.1	F: 5′-GTG CAG TCT GAA GTT CTC CT-3′ R: 5′-TTT CCA AAA CCT GTC TTC GG-3′	52	qRT–PCR	-	181	-
*ADH1C*	XM_004009680.4	F: 5′-GAA TCT GTC GCT CAG ATG AC-3′ R: 5′-GCT CAT TCA GGT CGT GTT TC-3′	52	qRT–PCR	-	225	-
*OLFML3*	XM_004002351.5	F: 5′-TCC AGA GTA GTG AGA GAG AC-3′ R: 5′-ACA AAA GGA ACA AGA TCA GC-3′	53	qRT–PCR	-	182	-
*THOC5*	XM_042234811.1	F: 5′-ATT GGC CCA CAT CAG GTT GA-3′ R: 5′-TCT CCC ATG GTG ACT TCT GC-3′	53	qRT–PCR	-	237	-
*CYP2E1*	NM_001245972.1	F: 5′-ATT CCC AAG TCC TTC ACC AG-3′ R: 5′-GTT GTT TTT GTG CAC CTG GA-3′	61	qRT–PCR	-	180	-
*LPIN1*	NM_001280700.1	F: 5′-CTC AGA CCA TGA ACT ACG TC-3′ R: 5′-AGT TTC ATG TGC AAA TCC AC-3′	57	qRT–PCR	-	247	-
*FABP5*	NM_001145180.1	F: 5′-GTC TGC AAC TTT ACG GAT GG-3′ R: 5′-CAG CAG TAT GGA GAT TTG CT-3′	61	qRT–PCR	-	233	-
*GAPDH*	NC_019460.2	F: 5′-GAG AAA CCT GCC AAG TAT GA-3′ R: 5′-TAC CAG GAA ATG AGC TTG AC-3′	62	qRT–PCR	-	203	-
*β-Actin*	NC_019471.2	F: 5′-GAA AAC GAG ATG AGA TTG GC-3′ R: 5′-CCA TCA TAG AGT GGA GTT CG-3′	62	qRT–PCR	-	194	-
*ANGPTL2*	NC_040254.1	F: 5′-ACA GCT CTG CTC TTA GGA GA-3′ R: 5′-AGA AGC TAG GGA ATC TTG CC-3′	62	Genotyping	*NsbI*	454	GG: 154, 300 bp AA: 454 bp GA: 154, 300, 454 bp
*OLFML3*	NC_019458.2	F: 5′-ATG ATG GCT ACC AGA TTG TC-3′ R: 5′-AGT CTG CAG TAC AGA AGG AG-3′	59	Genotyping	*MspI*	498	CC = 195, 303 bp TT = 498 bp CT = 195, 303, 498 bp
*THOC5*	NC_019474.2	F: 5′-CCC AGG AAG GTT TGA TTC TC-3′ R: 5′-AGG ACT ACA TGG TAG GTG TG-3′	60	Genotyping	*TaiI*	322	CC = 129, 193 bp TT = 322 bp CT = 129, 193, 322 bp

Tm: melting temperature, bp: base pair.

**Table 3:** Summary of hepatic sequence reads alignment to sheep reference genome.

Group	Sample	Total Number of Reads (Million)	Unmapped Reads (Million)	Mapped Reads (Million)	Percentage of Unmapped Reads (%)	Percentage of Mapped Reads (%)	Q20 (%)	Q30 (%)
**Low Tenderness**	LT1	20.95	2.67	18.28	12.74	87.26	96.48	92.68
	LT2	21.90	2.62	19.28	11.96	88.04	96.52	92.80
	LT3	20.06	2.40	17.66	11.96	88.04	96.06	91.95
	LT4	21.04	2.36	18.68	11.22	88.78	96.32	92.45
	LT5	20.84	2.48	18.36	11.90	88.10	96.45	92.67
**High Tenderness**	HT1	21.29	2.62	18.67	12.31	87.69	96.40	92.58
	HT2	20.00	3.23	16.77	16.15	83.85	96.50	92.70
	HT3	20.18	2.46	17.72	12.19	87.81	96.65	93.05
	HT4	20.02	3.12	16.90	15.58	84.42	96.41	92.56
	HT5	21.17	2.37	18.80	11.20	88.80	96.65	93.12

**Table 4:** Functional categories and corresponding genes that were over expressed in the liver tissues from sheep with high meat tenderness.

Category	Term	Count of Genes	Genes
Biological Process	Heart development	7	*ADM*, *KCNJ8*, *RPS6KA2*, *PDLIM3*, *GLI2*, *CACNA1C*, *DNAH5*
	Defense response to Gram-negative bacterium	3	*ADM*, *HMGB2*, *SSC5D*
	Cardiac muscle cell apoptotic process	2	*NOL3*, *RPS6KA2*
	Defense response to Gram-positive bacterium	3	*ADM*, *HMGB2*, *SSC5D*
	Positive regulation of vasculogenesis	2	*ADM*, *TMEM100*
	Odontogenesis of dentin-containing teeth	3	*HAND2*, *SOSTDC1*, *GLI2*
	Negative regulation of cardiac muscle cell apoptotic process	2	*NOL3*, *HAND2*
	Negative regulation of oxidative stress-induced intrinsic apoptotic signaling pathway	2	*NOL3*, *VNN1*
Cellular Component	Extracellular matrix	5	*OGN*, *AEBP1*, *EFEMP1*, *SSC5D*, *LOXL1*
	Extracellular space	16	*F11*, *PLAT*, *AEBP1*, *ADAMTS13*, *EFEMP1*, *HMGB2*, *POMC*, *S100A13*, *TNFRSF9*, *OGN*, *ADM*, *SOSTDC1*, *REN*, *GDF10*, *ANGPTL1*, *SSC5D*
	Proteinaceous extracellular matrix	5	*BGN*, *ADAMTS13*, *COL6A1*, *PRELP*, *WNT2B*
	Extracellular exosome	32	*AEBP1*, *CSPG4*, *ALDH1L2*, *EXTL2*, *CXCL12*, *OGN*, *ASPA*, *TGM3*, *COL6A1*, *VNN1*, *ANGPTL1*, *ANGPTL2*, *RHOF*, *RAP2B*, *PLAT*, *F11*, *DDC*, *FAM26E*, *AK1*, *EFEMP1*, *ACTN2*, *REEP2*, *S100A13*, *LIN7A*, *PRELP*, *REEP5*, *BGN*, *CPE*, *FBLN7*, *ZNF114*, *PCYOX1*, *CDH11*
	Sarcolemma	3	*BGN*, *COL6A1*, *CCDC78*
Molecular Function	Oxidoreductase activity, acting on paired donors, with incorporation or reduction of molecular oxygen, reduced flavin or flavoprotein as one donor, and incorporation of one atom of oxygen	3	*CYP2D14*, *CYP2D14-like*, *CYP2E1*
	Oxidoreductase activity, acting on the CH-NH_2_ group of donors, oxygen as acceptor	2	*LOXL3*, *LOXL1*
	Calcium ion binding	11	*NOL3*, *SCUBE2*, *NOTCH4*, *EFEMP1*, *MYL1*, *FBLN7*, *TGM3*, *ACTN2*, *FKBP10*, *S100A13*, *CDH11*
	Iron ion binding	5	*P3H3*, *CH25H*, *CYP2D14*, *CYP2D14-like*, *CYP2E1*
	Copper ion binding	3	*LOXL3*, *LOXL1*, *S100A13*
	Glycosaminoglycan binding	2	*BGN*, *ENG*
	Scavenger receptor activity	3	*LOXL3*, *SSC5D*, *SCARA5*

**Table 5:** KEGG pathway corresponding genes that were found to be overexpressed in liver tissues from sheep with high and low meat tenderness.

Function	Number of Genes	Benjamini-Hochberg *p*-Value	Genes
Ascorbate and aldarate metabolism	3	0.025004	*UGT2B18-like*, *UGT2B31-like*, *UGT2A1-like*
Pentose and glucuronate interconversions	3	0.045079	*UGT2B18-like*, *UGT2B31-like*, *UGT2A1-like*
Porphyrin and chlorophyll metabolism	3	0.066439	*UGT2B18-like*, *UGT2B31-like*, *UGT2A1-like*
Drug metabolism—other enzymes	3	0.066439	*UGT2B18-like*, *UGT2B31-like*, *UGT2A1-like*
Renin secretion	4	0.029158	*REN*, *GUCY1B2*, *CACNA1C*, *LOC101116002*
Retinol metabolism	4	0.030325	*UGT2B18-like*, *UGT2B31-like*, *ADH1C*, *UGT2A1-like*
Rheumatoid arthritis	4	0.089781	*MMP1*, *DQA*, *CXCL12*
Serotonergic synapse	4	0.095866	*DDC*, *CYP2D14*, *CYP2D14-like*, *CACNA1C*
Drug metabolism—cytochrome P450	5	0.003294	*UGT2B18*, *UGT2B31-like*, *ADH1C*, *UGT2A1-like*, *CYP2E1*
Metabolism of xenobiotics by cytochrome P450	5	0.004904	*UGT2B18-like*, *UGT2B31-like*, *ADH1C*, *UGT2A1-like*, *CYP2E1*
PPAR signaling pathway	5	0.005732	*MMP1*, *PLIN1*, *APOA5*, *ACSL6*, *FABP5*
Chemical carcinogenesis	5	0.007657	*UGT2B18-like*, *UGT2B31-like*, *ADH1C*, *UGT2A1-like*, *CYP2E1*
cGMP-PKG signaling pathway	5	0.099557	*KCNJ8*, *GUCY1B2*, *MRVI1*, *CACNA1C*, *MYL9*
Steroid hormone biosynthesis	7	8.33 × 10^−5^	*UGT2B18*, *UGT2B31*, *DHD3-like*, *UGT2A1*, *CYP2D14*, *CYP2D14-like*, *CYP2E1*

**Table 6:** Genotypes and association studies of selected candidate meat quality markers.

Meat Quality	*OLFML3* C > T			*ANGPTL2* G > A			*THOC5* C > T		
	Genotype (µ ± S.D)			Genotype (µ ± S.D)			Genotype (µ ± S.D)		
	CC (*n* = 57)	CT (*n* = 62)	TT (*n* = 21)	GG (*n* = 21)	GA (*n* = 69)	AA (*n* = 50)	CC (*n* = 135)	CT (*n* = 3)	TT (*n* = 2)
pH value	6.08 ± 0.58	5.93 ± 0.56	5.83 ± 0.54	6.09 ± 0.75	5.98 ± 0.61	6.06 ± 0.55	6.01 ± 0.62	5.97 ± 0.10	5.90 ± 0.41
Tenderness (shear force, kg/cm^2^)	**3.63 ± 0.91 ^ab^**	**3.79 ± 0.67 ^a^**	**3.35 ± 0.44 ^b^**	**3.09 ± 0.51 ^a^**	**3.61 ± 0.74 ^a^**	**3.75 ± 0.86 ^b^**	**3.55 ± 0.70 ^b^**	**4.97 ± 0.53 ^a^**	**3.45 ± 1.20 ^b^**
Cooking loss (%)	**45.31 ± 8.53 ^b^**	**46.47 ± 7.76 ^ab^**	**49.54 ± 7.35 ^a^**	49.47 ± 5.90	46.36 ± 8.09	46.85 ± 8.01	46.44 ± 8.05	48.42 ± 3.00	49.69 ± 3.88
WHC (mgH_2_O)	84.80 ± 12.00	83.88 ± 8.02	84.07 ± 6.98	84.04 ± 7.36	84.59 ± 9.37	84.16 ± 10.48	84.18 ± 9.53	77.95 ± 11.30	84.87 ± 4.32
WHC (% mgH_2_O)	28.26 ± 4.00	27.96 ± 2.67	28.02 ± 2.32	28.01 ± 2.45	28.19 ± 3.12	28.05 ± 3.49	28.06 ± 3.17	25.98 ± 3.76	28.29 ± 1.44

µ: mean, S.D: standard deviation, ^a,b^ Means differ significantly (*p* < 0.05) in the same row with different superscripts. The numbers in parentheses represent the number of individuals who have the specified genotype (Duncan’s test).

## Data Availability

The data were submitted to the database of the NCBI. The accession number is PRJNA847713, ID: 847713.

## Supplementary Materials

The following supporting information can be downloaded at: https://www.mdpi.com/article/10.3390/ani13040674/s1, Figure S1: Garut composite sheep (GCS) mating scheme. M = Moulton Charollais, G = Garut, H = St. Croix; Table S1: Differentially expressed hepatic genes in Garut composite sheep with divergent meat tenderness; Table S2: Top 30 up and down regulated hepatic genes obtained from sheep with high and low meat tenderness; Table S3: List of seed genes of the hepatic PPI network of hepatic transcriptomes from Garut composite sheep with their centrality measures; Table S4: List of seed genes for co-expression network; Table S5: Total SNPs detected by RNA-Seq in hepatic genes in Garut composite sheep divergent meat tenderness; Table S6: Polymorphisms detected in the highly polymorphic hepatic DEGs; Table S7: Genotype, allele frequencies and the chi-squared test of selected SNPs validated using RFLP.
