# Supplementary figures and images for: Hepatic Transcriptome Analysis Reveals Genes, Polymorphisms, and Molecules Related to Lamb Tenderness
Source: Animals (Basel). 2023 Feb 15;13(4):674. doi: 10.3390/ani13040674 (PMC9951696; doi:10.3390/ani13040674)

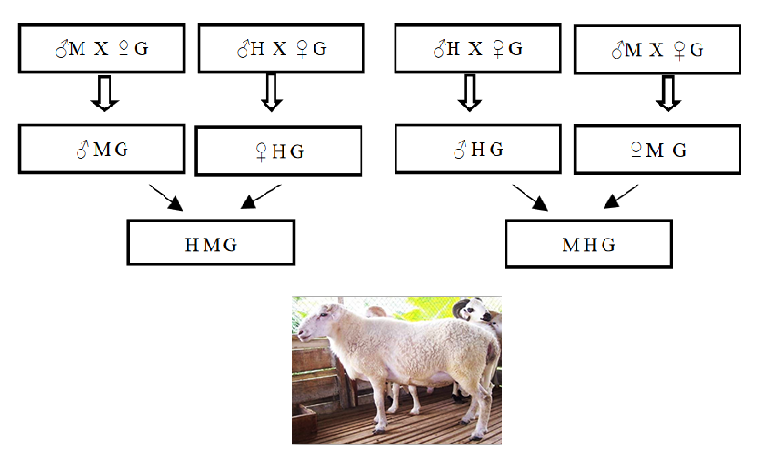

Supplement: Supplementary file 1 [file animals-13-00674-s001.zip › Supplementary Files New/Figure S1 - Garut composite sheep (GCS) mating scheme.tif]
